# Supplementary material for: Opposing Roles of S1P3 Receptors in Myocardial Function
Source: Cells. 2020 Jul 24;9(8):1770. doi: 10.3390/cells9081770 (PMC7466142; doi:10.3390/cells9081770)
Supplement: Supplementary file 1 [file cells-09-01770-s001.pdf]

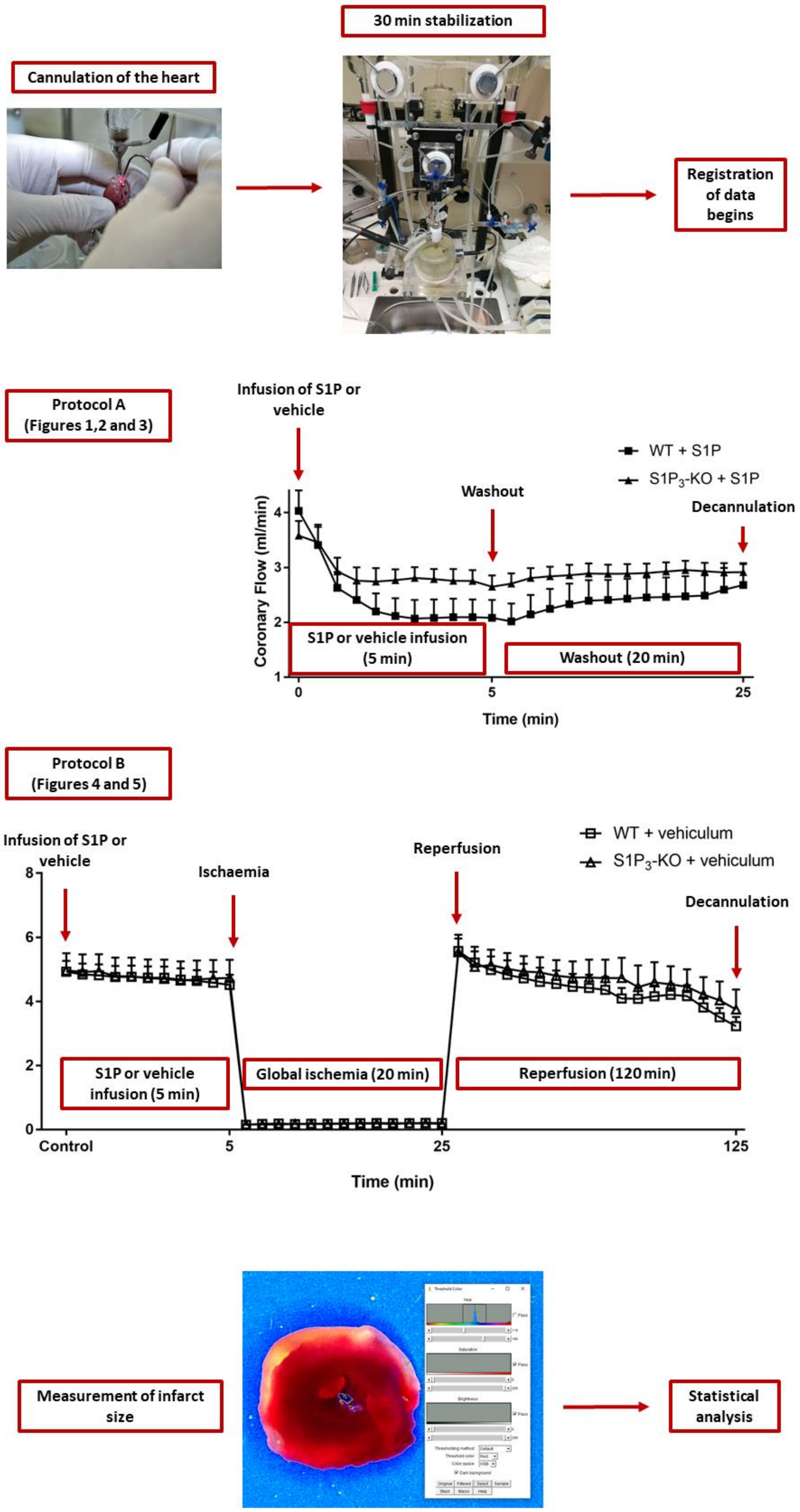

30 min stabilization
Cannulation of the heart
Registration of data begins
Protocol A (Figures 1,2 and 3)
Infusion of S1P or vehicle
WT + S1P
$S1P_3$-KO + S1P
Washout
Decannulation
Coronary Flow (ml/min)
S1P or vehicle infusion (5 min)
Washout (20 min)
Time (min)
Protocol B (Figures 4 and 5)
Infusion of S1P or vehicle
Ischaemia
Reperfusion
WT + vehiculum
$S1P_3$-KO + vehiculum
Decannulation
S1P or vehicle infusion (5 min)
Global ischemia (20 min)
Reperfusion (120 min)
Control
Time (min)
Measurement of infarct size
Statistical analysis

Suppl. Figure 1. Experimental protocols of isolated heart experiments. After cannulation, a 30-min stabilization period was allowed. Subsequently, baseline data were recorded and S1P or vehicle was infused to the perfusion line for 5 min. Afterwards, depending on the experiment, either a 20-min washout period (Protocol A) or a 20-min global ischemia caused by complete cessation of perfusion was applied. At the end of the ischemic period, perfusion was re-introduced and reperfusion was followed up for 2 h (Protocol B).

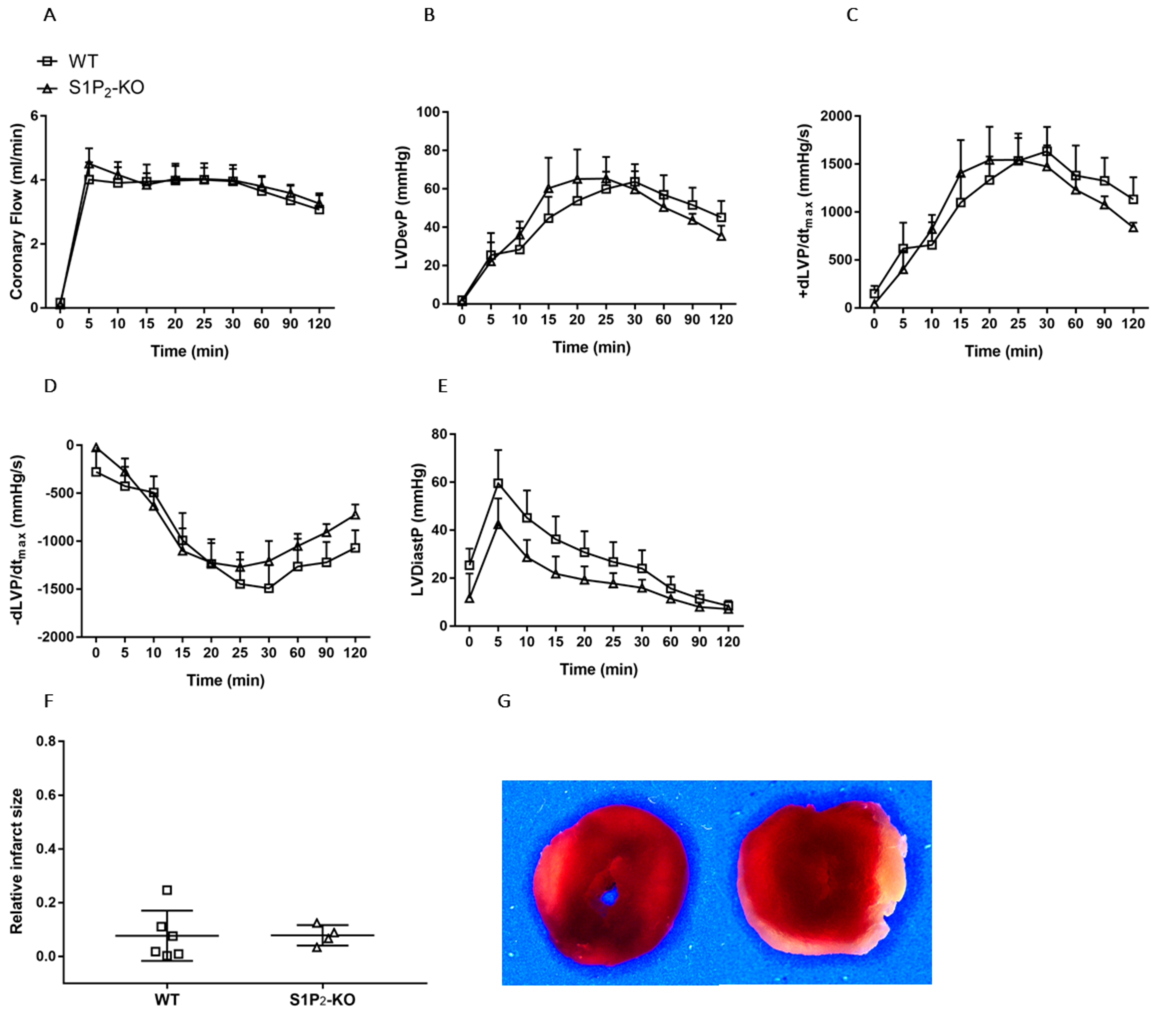

Suppl. Figure 2. Postischemic coronary flow (CF) (A), left ventricular developed pressure (LVDevP) (B), +dLVP/dtmax (C), -dLVP/dtmax (D) and left ventricular diastolic pressure (LVDiastP) (E) in isolated WT and $S1P_2$ knock-out (KO) mouse hearts. Vehicle was administered for 5 min to the perfusate before the induction of a 20-min ischemia followed by a 120-min reperfusion period. Relative infarct size (F) and representative sections (G) from hearts subjected to ischemia/reperfusion. Mean ± SEM; n=6, 4; statistics: two-way repeated measurement ANOVA and Dunnett's *post hoc* test (A-E) and unpaired *t*-test (F).
